# Does commerce promote theft? A quantitative study from Beijing, China

**DOI:** 10.1057/s41599-023-01706-x

**Published:** 2023-05-05

**Authors:** Yutian Jiang, Na Zhang

**Affiliations:** 1 Department of Economics, School of Economics and Management, Beijing Jiaotong University, Beijing, China; 2 Beijing Laboratory of National Economic Security Early-warning Engineering, Beijing Jiaotong University, Beijing, China

**Keywords:** Criminology, Environmental studies

## Abstract

Commerce, as both an environmental and a social factor, is essential to the study of the causes of urban crimes. This paper aims to comprehensively propose research hypotheses based on these two types of commercial factors and optimise statistical tools with which to analyse commerce’s impact on the level of theft in Beijing. Combining criminal verdicts, census data, points of interest, and information on nighttime lighting, this paper first applies a hierarchical regression model to verify the effectiveness of using commercial environmental and social factors to explain theft statistics and then constructs a structural equation model to analyse the joint influence of multiple commercial factors on those statistics. This paper finds that Beijing’s commerce does not significantly promote theft, verifies the effectiveness of two types of commercial variables and the corresponding Western theories in explaining commerce’s impact on theft in Beijing, and provides empirical data for the study of the causes of theft in a non-Western context.

## Introduction

In contemporary China, urbanisation has caused the population to rapidly concentrate in large cities (Lu, 2016), thus promoting the aggregation of social activities such as crime, which undoubtedly exacerbates the vulnerability of urban security. Park (1915), the founder of the Chicago School, advanced the classic topic that “Crime is the problem of the city.” Currently, there is far more crime in large cities than in small cities or rural areas, and this crime is more violent (De Nadai et al., 2020). In Chinese urban crimes, thefts are of particular concern. First, theft accounts for an extremely high proportion of all crimes. From 1978 to 2019, theft ranked first among all criminal cases filed by public security forces. In 2020, theft ranked second only after property fraud (affected by the depressed economic environment during COVID-19, this type of crime, represented by telecom fraud, accounted for a 10.58% surge^1^). It can be seen that the theft problem is severe and has not been fundamentally addressed. At the same time, theft generally arises from a temporary intention and is thus more susceptible to the influence of the surrounding environment, so spatial prevention and control measures can be used to effectively suppress it (Shan, 2020). In summary, it is necessary to conduct spatial research on theft in Beijing to effectively govern the growing theft problem, reduce governance costs, maintain urban stability, improve residents’ happiness, and provide a reference to be used by other major cities in China to solve their own theft problems.

In studying the causes of urban crime such as theft, commerce^2^ deserves special attention. First, commerce is an essential part of urban functions in the Athens Charter, and commercial or economic conditions are common and major causes of theft that need to be studied. Second, the dual economic structure is an unavoidable and vital phenomenon in China. Urban and rural products or economies are isolated and therefore evolve separately to a certain extent and have their own characteristics. Thus, developed commerce and a prosperous economy distinguish cities from rural areas, so studying commerce is particularly important for understanding urban crimes. Since commerce is both an environmental and a social factor, it should be comprehensively characterised by these two types of commercial factors, and its impact on thefts should be analysed using various theories.

Based on the existing research, this paper aims to effectively explain the impact of commerce on thefts through reasonable hypothesis construction and optimised statistical tests (Weisburd et al., 2014) to verify whether Western-derived theories such as environmental criminology and social disorganisation theory (SDT) apply in a non-Western settings (Piquero et al., 2019) and to provide this field with an eastern empirical study conducted in Beijing, China. First, this paper uses correlation analysis and a hierarchical regression model to verify the applicability and effectiveness of the two types of commercial factors in explaining the impact of commerce on thefts in Beijing. On this basis, this paper constructs a structural equation model that can reflect the primary spatial relationships and analyses the joint influence of multiple commercial factors on thefts with the help of the latent variable of commerce so that the relationship between commerce→theft is more intuitive.

The research significance of this paper is mainly reflected in the following two points: (1) By studying cases of theft, which have been judged to be criminal offences and account for a very high proportion of the total crime in Beijing, this research is meant to provide insight into the impact of commerce on theft in Beijing, providing a reference for the prevention and control of urban theft and providing a basis for protecting the happiness of urban residents (Cheng and Smyth, 2015). (2) Criminological research is more highly developed in the West. For example, environmental criminology research focuses on European and American cities by applying Western perspectives (Musah et al., 2020). Due to the extreme lack of statistics at the community level and the fact that most criminologists come from a legal background and lack sociology skills, Chinese SDT-based research still faces difficulties, such as innovating data sources and overcoming the challenges of combining interdisciplinary theories. This is why the total amount of such research is still small. Expanding the research scope to investigate new situations can provide more cases for research in these fields. By studying the theft problem in Beijing, a Chinese megacity, the feasibility of influencing factors impacting the conclusions of Western research in solving the same issues in China can be tested and whether these factors can be manipulated to guide the formulation and implementation of prevention and control measures in large cities in China can be explored.

The main innovative contributions of this paper are as follows: In addition to providing oriental empirical data, this paper selects two statistical models that complement each other and most closely match the dual characteristics of commerce. Ideal statistical models are applied to verify the accuracy of the research assumptions and variable selection, assure consistency with realities and statistical requirements, and facilitate the study of the combined impact of commerce on thefts due to commerce being both environmental and social in nature. The hierarchical regression model can be used to highlight the optimal choice of commercial environmental and social variables, to prove the validity and applicability of the two by placing these two types of variables at different levels, and to verify certain research hypotheses, their construction method and the partial results of the structural equation model. The structural equation model can not only depict realistic spatial relationships and meet the statistical requirements, but it can also analyse the comprehensive impact of the two types of commercial variables on theft through the application of the latent variable, making the relationship between commerce and theft intuitively visible. By selecting statistical models that are more compatible with the dual characteristics of commerce, this paper better analyses the comprehensive impact of commerce on theft in Beijing and provides ideas for future researchers.

## Literature review

### Influencing factors of theft

Existing studies have multiple interpretations of the spatial distribution characteristics of theft, covering both the environmental and social factors in play at the place where theft occurs. In the Residential Theft Study, Wu et al. (2015) found that street network structure and socioeconomic factors were dual factors influencing residential theft and drew some important conclusions, such as high street permeability can dampen theft and commerce has a positive correlation with theft. Chen et al. (2017) studied the impact of sociodemographic factors on residential theft. They found that factors such as the proportion of the population who rent their houses and the proportion of residents who were originally from other provinces have a positive effect on the number of residential thefts and that factors such as the percentage of residents with a bachelor’s degree or higher are negatively correlated with the number of residential thefts when controlling for some factors in the bussing system. Xiao et al. (2018) found that the distance of crime in residential theft cases was closely related to the characteristics of the home community and the target community. They found that after searching for crime targets in and near their home community, perpetrators then decide whether to commit crimes at a longer distance based on factors such as the wealth of the target community. In a general theft study, based on social disorganisation theory and routine activity theory (RAT), Liu and Zhu (2016) argued that community social characteristics largely influence the incidence of theft. They found that communities with higher-than-average bar and department store density have a higher risk of theft. Yue et al. (2018) found that a range of factors related to socioeconomic and communal facilities impacted thefts and validated this effect with sociodemographic data and facility data. They found that higher local permeability ensures better street safety, while higher nonlocal permeability poses a threat to security. At the same time, Mao et al. (2018) found that Shanghai vehicle theft occurs in stable crime hotspots. They suggested that highly mobile and short-lived populations, higher population densities, and more traffic flows all positively impacted motor vehicle thefts. These findings also support routine activity theory. It can be seen from these observations that there are many factors that influence theft, and the focus of existing studies also varies, so researchers should firmly make the choices and trade-offs among research factors that are needed in this field. Among them, commercial or economic factors are the most common and major research factors. Scholars have used a variety of datasets to measure their impact on theft from different perspectives.

### Commerce and thefts

Commerce could be seen as an environmental factor. Related research explains the relationship between commercial environmental factors and theft with the help of rational choice theory (RCT) and RAT. RCT is an essential foundational theory of environmental criminology that posits that potential perpetrators weigh the potential benefits and consequences of theft and then rationally judge whether to commit the theft. On this basis, RAT proposes that theft can occur when potential offenders, suitable criminal targets, and incompetent supervision converge at a particular crime location. In environmental criminology, a basic consensus is that drinking places and other undesirable enterprises can increase the crime rate regarding theft (Liu and Zhu, 2016). Yu and Maxfield (2014) studied the impact of ordinary enterprises on commercial and residential theft (but their research results only pertain to commercial theft and not residential theft). They found that ordinary enterprises also increase the risk of theft because these harmless or ordinary places can expose targets to the criminal population. Sohn (2016) studied the relationship between commercial land use and residential theft and found that the relationship between the two is relatively complex. For example, the impact of commercial land use on residential theft varies by the type of commercial facilities, and not all commercial uses increase crimes in the area. That is, an increase in specific commercial land use may either increase or decrease crime. Based on this finding, this paper suggests that when commercial facilities that encourage legitimate activities are incorporated into the community, they can offset the opportunity effect of attracting offenders by enhancing the positive effect of surveillance. According to the regression analysis results, Mao et al. (2018) found that the number of commercial premises in an area has a restraining effect on motor vehicle theft cases in that area. They first argued that this result is contrary to common sense since “areas with more commercial premises have more potential targets for crime” but after empirical investigation they finally attributed this phenomenon to the idea that areas with more commercial premises also having better and more orderly area management. The relationship between commercial environmental factors and theft is complex and requires extensive verification, especially in non-Western settings.

At the same time, commerce can also be seen as a social factor. As SDT puts it, social factors are important determinants of crimes. Crimes are caused by people’s natural reactions to society. SDT explains the impact of society on crimes, with a strong emphasis on commercial or economic factors. This theory holds that economic growth and commercial prosperity have profoundly changed the social life of urban residents, driving social problems such as economic inequality and population migration and giving rise to crimes such as theft (Chen et al., 2021). Accordingly, Wu et al. (2015), Chen et al. (2017), Xiao et al. (2018), Liu and Zhu (2016), Yue et al. (2018), and Mao et al. (2018) all introduced social factors such as economy, population, and employment to explain the theft phenomenon. Using measures such as population mobility, housing stability, ethnic consistency, and urbanisation to capture neighbourhood characteristics, the relevant research attempts to introduce the structural characteristics of neighbourhood society to analyse crime causes. Most extant environmental criminology studies focus on the environmental factors of the places where thefts occur and introduce social factors to improve the accuracy of spatial theft analysis, thus comprehensively analysing the theft problem, which is a problem that is closely related to society and the environment simultaneously (Tang et al., 2019). This is a valuable exploration, but due to the limited social data sources at the community level in China, innovative and careful use of unofficial data is vital.

To address this issue, nighttime light data is used in this study to reflect commerce prosperity at the township level. In traditional criminological research, scholars have verified that nighttime lights can prevent crimes, and most of their studies have linked nighttime lights with street lighting as a microscopic measure (Welsh et al., 2022). Some recent studies have increasingly used nighttime data, such as nighttime lights or nighttime social media, to represent social factors. For example, Zhang et al. (2021) found that using nighttime social media data to measure burglary victims worked well, and that these data worked best in the early hours of the morning. They demonstrated that the use of daytime active population data did not explain burglary effectively, while the use of nighttime data had strong interpretive power. Based on the hypothesis advanced in crime pattern theory that edges affect crime, Liu et al. (2020) and Zhou et al. (2019) studied the compound edge effect. They verified the existence of compound edges and their effects on crime and proved that using nighttime light data to measure compound edges effectively improved model fit. Nighttime light data are crucial for understanding compound edges, including physical and social edges, and have the potential to represent social factors. In economics research, nighttime light data are often used to represent regional economic situations. Economists have verified the validity of nighttime light data in representing macro- and meso-economic conditions, summarising the shortcomings of these data in reflecting the economic situations of low-density cities or rural areas and their corresponding microscopic lighting differences and advantages in high spatial accuracy, addressing the lack of GDP and being suitable for scales as fine as one square kilometre (Gibson, 2021; Gibson et al., 2020; Gibson et al., 2021). Therefore, this paper uses nighttime light data to represent the commerce prosperity of Beijing, a high-density developed city, at the meso township level (1.09 to 84.5 km^2^) and uses fine data to measure the commercial social factors and capture the characteristics of areas where crime occurs more accurately through the use of innovative data sources (Snaphaan and Hardyns, 2021).

### Other factors and thefts

In addition to commerce, this paper examines the impact of transportation, police agencies, and population factors on theft.

According to the Athens Charter, the four functions of a city are residence, recreation, work and transportation. Commerce can reflect the recreation and work activities of city dwellers. Therefore, this paper introduces transportation factors with which to comprehensively evaluate the urban environment. Among these transportation factors, road accessibility, bus station location and density, number of bus routes, and traffic flow are often studied. For example, Liu et al. (2017) found that adding a one-way bus line can significantly decrease burglary throughout a region and other developed regions but does not have a statistically significant impact on burglary in developing regions. Chen et al. (2017) verified that bus routes and bus stop density exert a significant positive effect on burglary based on rational choice theory, as bus routes improve the accessibility of the area so that criminals can come and go freely and increase the levels of criminal motivation. Nevertheless, at the same time, they also illustrate that some studies have found that inaccessible areas may be more vulnerable. The relationship between public transportation and theft in China needs more extensive empirical research. Commerce and transportation also interact with each other. Using correlation analysis, Porta et al. (2012) studied the relationship between street distribution and economic activities in Barcelona, Brazil. They found that the spatial distribution of secondary economic activities, that is, the distribution of commercial activities such as retail commerce, hotels, restaurants, and cafes, is highly correlated with the distribution of streets. However, other commercial activities that are unrelated to the public have a low correlation with the spatial distribution of streets. This study suggested that secondary economic activities are more dependent on passers-by and are therefore more limited by their locations, while major economic activities make people more willing to travel to them through the attractiveness of their functions. The impact of transportation on theft and its interrelationship with commerce is analysed in this paper.

Police agencies are also important research subjects. Liu and Zhu (2016) and Liu et al. (2019) explain that the nonsignificant or positive relationship between policing factors and theft is due to the fact that the enhancement of policing is a natural response to high crime rates. Shan (2020) found that the crime containment effect of government agencies and police agencies is relatively limited near large commercial places because shopping malls attract crime and the spatial layout of institutions is difficult to crime hotspots in a timely manner. Blesse and Diegmann (2022) found that closing police stations increased car thefts and burglary cases because this practice reduced police visibility and criminal deterrence. Piza et al. (2020) found that police stations increase the visibility of police, thus creating a deterrent effect within the precinct. They also find that the substation’s role could be strengthened with increased policing activities.

In terms of population, concepts such as hukou, resident population, floating population, etc., complicate the Chinese population issue. The hukou is a legal document in China that records household population information and is used to regulate population distribution and migration. Resident population describes those residents who have lived in a household for more than 6 months, including both the resident population with hukou and the resident floating population who have lived for more than 6 months in the area. The floating population refers to the resident population that can legally work for a long time but lack certain rights in public services, education, and medical care compared to those with hukou. Jing et al. (2020) found that when people do not have local hukou, they feel a greater sense of social disorder, less social integration, and a greater fear of crime. Chen et al. (2017) demonstrated that the proportion of the population that rents their houses and the population of other provinces promote residential theft and that the proportion of the population with an educational level of bachelor’s degree or higher inhibits residential theft. The SDT explains these findings. Residential mobility leads to weak local social connections, low community belonging, and weak oversight and guardianship capacity, which further weakens informal social control networks and ultimately causes crime. As Beijing’s population density ranks high in China and the hukou is designed to regulate population distribution, the proportion of the floating population in Beijing’s resident population is high. At the same time, Beijing is China’s higher education and cultural centre, and the proportion of higher educated people in the floating population is high. Accordingly, this paper argues the following: (1) The resident population of each township officially announced by Beijing Municipality suitably reflects the neighbourhood characteristics related to crime. That is, it is ideal for explaining thefts. (2) Beijing’s resident population (including both the local population with hukou and the resident floating population) feels a lower level of social disorder and a higher level of social integration, which may discourage thefts.

In summary, scholars have addressed the environmental and social factors affecting thefts in the existing research. Among them, commerce, transportation, police agencies, and population factors are vital. Due to the differences between the national conditions of China and those of the West, there are specific differences between Chinese and Western studies. For example, Western studies often examine population heterogeneity in terms of race and immigration. Nevertheless, Chinese studies see the resident population and the population with hukou as essential indicators. China’s transportation system is more complex (Liu et al., 2017), so some Western findings may not apply to China. Therefore, this paper evaluates the impact of commerce on theft in Beijing, verifies the effectiveness and applicability of commercial environmental and social factors in explaining the relationship between commerce and theft in China, and provides Chinese empirical data for the study of theft causes.

## Research design

### Research objective and research method

Commerce is an important object in studying the causes of urban crimes. With the vigorous development of commerce in Beijing’s main urban area, exploring the relationship between commerce and theft is particularly important to understanding the theft phenomenon. This paper divides commerce into commercial environmental and commercial social factors, aiming to comprehensively evaluate the commercial situation in Beijing and thoroughly analyse the impact of commerce on thefts. After fundamental statistical analysis and geographical portrait research, this paper mainly applies hierarchical regression and structural equation models to achieve the above research objectives.

In the hierarchical regression model, other factors, commercial environmental factors and commercial social factors, can be placed at different levels, highlighting the comprehensive impact of multiple factors on theft at different levels, helping to identify the different roles of various factors in explaining the theft phenomenon, and facilitating this capacity of this study to judge the impact of multiple factors on the theft phenomenon. Accordingly, the effectiveness of these two variables in analysing the impact of commerce on thefts can be verified.

The structural equation model has two main advantages. First, compared with the multiple regression model, the structural equation model can be used to portray the complex relationships between various variables to better align the with reality. Therefore, considering the complex interrelationships between spatial factors, a structural equation model is necessary. Second, the structural equation model can introduce a potential variable, such as restaurants, to represent commercial factors without directly using data. Therefore, this model has lower requirements for data characteristics, and it compensates for some of the disadvantages of traditional methods such as path analysis and negative binomial regression. The structural equation model also has some disadvantages, however. First, the structural equation model, although suitable for predicting dependent variables and verifying the established theoretical model, is a more demanding theoretical model with more demanding research assumptions. This is why this paper explores its topic using a hierarchical regression model before applying structural equation models, aiming to ensure the correctness of certain assumptions and to test the necessity of introducing these two types of factors. Second, due to computational limitations, the structural equation model assumes that all relationships are linear (Najaf et al., 2018). For this paper, the latent variables that play an essential role in the structural equation models can be used to infer the overall nature of factors that are difficult to directly represent with one data point, such as commerce, based on multiple observation variables, to measure the relationships between this latent variable and other variables, and to make the relationship between commerce and theft that is the focus of this paper more intuitively visible (Skrondal and Rabe-Hesketh, 2007; Lee and Song, 2014).

In summary, given the high compatibility of hierarchical regression and structural equation models with the study’s purpose, this paper first applies the hierarchical regression model to analyse the applicability of commercial environmental and social factors in explaining theft numbers in Beijing, exploring the correctness of certain assumptions. After that, this paper takes the structural equation model as its main method and uses a latent variable to highlight the relationship between the effect of commerce on theft.

### Research hypotheses

**Fig. 1:**
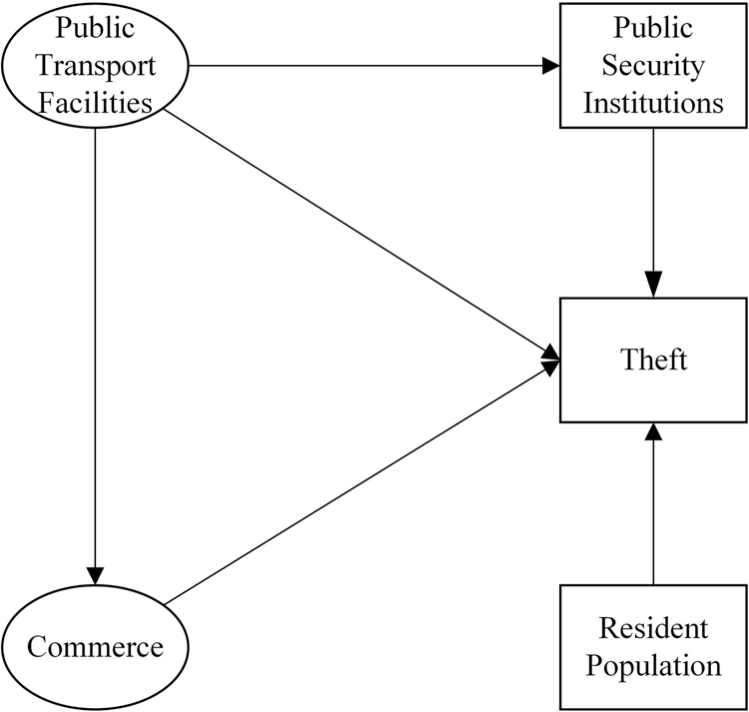
Conceptual analysis model. Commerce not only affects theft but also is affected by public transport, and public transport also affects theft and public security institutions. Theft is also influenced by public security institutions and permanent residents.

Based on the existing research results (Wortley et al., 2021) and the actual situation in China, this paper formulates the conceptual analysis model shown in Fig. 1. This model later supports the structural equation model, which helps to solve the dilemma of traditional regression models, such as their limited explanatory power and equal treatment of individual variables. Drawing on the ideas of economic modelling, this paper hopes to build a simple rather than complex and comprehensive conceptual model that depicts all aspects of the actual situation and highlights the main points. This paper aims to study the four types of influencing factors of theft and first introduces the relationships between the four types of factors and theft. Since public transport facilities are almost entirely planned and built by the government in China and should be regarded as an exogenous variable that exerts an essential impact on other factors, this paper introduces public transport facilities→commerce and public transport facilities→public security institutions as two pathways that are in line with reality and have been experimentally verified. Therefore, based on meeting the statistical requirements, this paper ensures realistic matching and model conciseness.

First, the number of public transportation facilities is an important indicator of the mobility of the population in a specific area, and it is also a common factor in the study of theft. According to the situation in China, areas with a greater number of public transportation facilities tend to receive more police forces to prevent potential crimes in these high-traffic areas. For example, the Beijing West Railway Station Substation, the Beijing West Railway Station Police Station, the Zhanbei Police Station, the Zhannan Police Station, and other police stations have been set up in proximity to the Beijing West Railway Station. The Beijing South Railway Station Police Station, Yangqiao Police Station, You’anmen Police Station, Xiluoyuan Police Station, and other police stations have also been set up in proximity to the Beijing South Railway Station. Given that public transportation facilities are mostly planned and constructed by the government and should be regarded as exogenous variables and that the distribution of public security agencies, including mobile police booths, police cars, and public security booths, is more flexible, the latter should be utilised as endogenous variables as they are affected by the former. In addition, limited by economic costs, the spatial layout of public security institutions, such as public security substations and police stations, cannot be easily changed, which limits their flexibility (Shan, 2020). This paper advances the claim that the distribution of police forces is difficult to flexibly change in time with the distribution of thefts, so a one-way relationship is drawn between the two. This paper follows the assumption that public transportation facilities affect thefts, public transportation facilities affect public security institutions, and public security institutions affect thefts in that area.

Second, the relationship between commerce and thefts can be explained by environmental criminology and SDT. The routine activity theory holds that crimes originate from daily life, and commercial premises undertake the two primary functions of urban areas, namely, recreation and work, which are closely related to people’s everyday lives. By providing food, clothing, entertainment, etc., commercial premises such as catering, shopping, and leisure are key nodes in the daily activities of both citizens and potential perpetrators and often see the highest incidence of theft because they limit the scope of people’s activities (Piquero et al., 2019). SDT values the commercial social factor of the nighttime light intensity and claims that the neighbourhood characteristic of commerce prosperity is closely related to crimes such as thefts. As far as the Beijing urban area is concerned, the different commerce prosperity of each township reflects its particular urban function. For example, the capital airport township, with the highest nighttime light intensity, is an important transportation hub busy around the clock. The Xiangshan township with the lowest intensity is a remote mountain and forest scenic area. Thus, their different levels of commercial prosperity mean that these townships’ urban functions and neighbourhood characteristics differ, leading to the different effects of informal social control on theft. As a result, commerce is often seen as an influencing factor on theft. Based on the research results and the current situation in China, this paper posits that a complex relationship exists between public transportation facilities and commerce (Shan, 2020; Porta et al., 2012). In China, public transportation facilities can be considered to be exogenous variables. Therefore, this paper sets the relationship between the two as a one-way relationship in which public transportation facilities affect commerce and emphasises that this relationship varies across the specific circumstances of each country.

Third, the population factor is essential to theft, and resident population density is suitable data for studying theft in Beijing (Jing et al., 2020; Chen et al., 2017). SDT attaches great importance to informal social control, which describes the informal and unofficial activity of regional inhabitants meant to fight crime through collective intervention. The higher that the density of the resident population in a township so, the less mobile the population, and the more likely the regional inhabitants are to pursue shared values and consciously unite to implement regulatory activities to maintain effective social control and combat theft. Therefore, this paper sets the resident population density, an important measure of informal social control, as the explanatory factor for theft.

## Study area, study variables, and data sources

### Study area

The urban area of Beijing, which includes Dongcheng District, Xicheng District, Chaoyang District, Haidian District, Fengtai District, and Shijingshan District, was divided into 134 townships in 2018 and is located in the south-central part of Beijing. Correspondingly, other areas are considered rural areas or suburbs. With the development of urbanisation, the difference between these six districts and their suburbs has become increasingly apparent. As the heart of Beijing, the urban area has a higher population density and greater theft numbers than other areas, becoming a virtual object of urban crime research. This paper aims to study the problem of theft in cities, so the urban area of Beijing is selected as the research object. Given that environmental criminology requires delicate spatial research, commercial social factors are challenging to measure at the micro level, and townships are the smallest administrative unit among the three levels of administrative divisions in China and the smallest announced unit for government plans and policies, such as police prevention and control policies, this paper explores the influence of the most fundamental commercial social factors and the commercial environmental factors that facilitate police deployment on theft numbers in Beijing at the township level, comprehensively analysing the relationship between commerce and theft.

### Study variables and data sources

In Table 1, the variables, data, and descriptive statistical indicators used in the study are summarised. The collinearity of all variables are checked by the variance inflation factor (VIF). The VIF values are mostly between 1.885 and 3.093; excepting the VIF value of the data regarding the densities of sports and leisure places at 6.445 and the VIF value of the data on the densities of catering places at 8.967, which indicates that there is no unacceptable significant correlation between these variables.

**Table 1:** Descriptive statistics of latent and observed variables.

Latent variables		Observed variables	*N*	Min	Max	*M*	SD	Kurt	Skew
Theft number (units)			134	0.00	39.00	8.69	6.97	3.84	1.70
Commerce	Nighttime light intensity (nanoWatts/cm^2^/sr)		134	3.66	80.88	31.29	12.44	1.43	0.47
	Commercial premises	Sports and leisure places’ density (units/km^2^)	134	0.94	308.28	53.27	51.47	7.29	2.22
		Shopping places’ density (units/km^2^)	134	5.56	3854.55	472.22	526.56	14.27	3.07
		Finance and insurance places’ density (units/km^2^)	134	0.44	367.27	46.65	59.89	9.51	2.77
		Catering places’ density (units/km^2^)	134	7.17	1838.18	360.58	337.85	3.55	1.69
Resident population density (units/km^2^)			134	0.42	62.68	13.86	9.81	3.56	1.17
Public security institutions’ density (units/km^2^)			134	0.00	10.09	1.69	1.78	6.96	2.29
Public transport facilities		Station density (units/km^2^)	134	0.60	23.64	8.91	5.09	−0.42	0.37
		Metro station density (units/km^2^)	134	0.00	10.00	1.69	1.75	3.49	1.63

*TD* Theft, *CI* Commerce, *NI* nighttime light intensity, *CF* commercial premises, *SL* sports and leisure places *SP* shopping places, *FI* finance and insurance places, *CA* catering places, *PD* resident population, *PS* public security institutions, *PT* public transport facilities, *BS* station, *MS* metro station, *N* sample size, *Min* minimum,*Max* maximum, *M* mean, *SD* standard deviation, *Kurt* Kurtosis, *Skew* skewness.

This paper examines those criminal cases of theft^3^ for which case data are readily available and the social impact is relatively severe, refers to the classification of ordinary theft, burglary, and the theft of motor vehicles as presented in the Law Yearbook of China, eliminates specific types of thefts and focuses on the study of ordinary theft. The numbers of thefts are calculated from the public judgement documents on the judgement document network, which is the official Chinese platform used to publish and provide all effective court judgement documents.

The process of collecting and processing the judgement document materials is as follows: (1) Based on the judgement document network, this work retrieves the criminal judgements on theft that were published from January 1, 2018, to January 15, 2022 and manually screens the judgements based on the conditions that the actual place of the crime is Beijing and the actual time of the crime (rather than time of publication of the documents) is 2018, resulting in a total of 3088 relevant judgement documents being retained. (2) This paper manually screens 3088 judgement documents on the condition that the crime actually occurred in the urban area of Beijing. Among them, 1751 documents were related to thefts in the urban area, accounting for 56.70%, while 1337 documents were only related to thefts in the remaining ten districts, accounting for 43.30%. Under the premise of a significantly smaller footprint, the urban area accounted for 56.70% of the total thefts in that year, which is in line with the classic statement that “crime is a problem of the city.” (3) Each location of theft as extracted from the judgement documents in 134 townships are identified in this study. In fact, most of China’s theft judgements specify the criminal address at least to the level of the township jurisdiction. This official uniform action facilitates the extraction and precise location of criminal addresses. Among them, 243 documents contain insufficiently detailed addresses for positioning, and 1508 documents provide sufficiently detailed addresses for positioning, thus enabling the successful location of 86.12% of the criminal theft offences in the six districts. (4) Of the 1,508 theft cases available for research, this paper classifies theft cases into the categories of ordinary theft, burglary and motor vehicle theft using the classification of the China Law Yearbook since 2010, which are in line with Chinese national conditions. 976 ordinary thefts are identified in this paper, accounting for 64.72% of the total. This proportion is similar to the 67.91% of ordinary theft criminal cases reported by public security forces nationwide in 2018, indicating the representativeness of this study sample. Finally, the number of ordinary thefts in each township are tallied. Among the crime addresses, 17 addresses describe bus thefts in the form of “the theft occurred on the way from Station A to Station B.” In this regard, this paper sets Stations A and B with a weight of 0.5. When a case involves multiple thefts, this paper counts the thefts at each location once and avoids double-counting thefts at the same location.

In terms of the choice of research period, careful consideration was made. First, given the objective existence of crime time hotspots, this paper, as a spatial study, tends to analyse thefts on a yearly basis. Second, given that the disclosure process of judgement documents takes a long time, the materials of judgement documents in too recent years are not applicable. On January 12, 2023, this paper retrieved criminal judgements on thefts in Beijing and obtained a total of 3752 criminal judgements in 2018, 3765 criminal judgements in 2019, 2449 criminal judgements in 2020, 1390 criminal judgements in 2021, and 389 criminal judgements in 2022, which shows that the numbers of judgements in later years are significantly lower. It is highly likely that the judgements for later years have not yet been fully disclosed and are not suitable for this study. After careful consideration, this paper finally uses the 2018 judgement documents for study.

The nighttime light data obtained from global satellite observations from VIIRS_VNL V2 are one of the academic community’s most widely used geospatial data products and have been verified to perform better when representing economic activities (Gibson et al., 2021). Based on the 2018 data and ArcGIS software, this paper calculates the average nighttime light intensity of 134 townships in 2018.

The numbers of commercial premises, public security institutions, and public transportation facilities are tallied from the POI data provided by Amap. This paper uses POI data from 2018, which has been continuously updated until the end of 2018 and filters out discontinued or deactivated points of interest. Based on these data, the various POI data of 134 townships are extracted and counted. Specifically, among commercial premises, this paper first studies shopping places and catering places because these two types of places are closely related to people’s lives. Second, this paper also studies two types of commercial premises with high risks of theft, namely, finance and insurance establishments and sports and leisure establishments. Sports and leisure establishments include undesirable commercial premises such as bars and nightclubs, which are often of concern in environmental criminology. As it is difficult to distinguish between a sports or leisure category for certain commercial establishments, such as roller skating rinks, dance halls, and golf clubs, this paper combines these two types of places to avoid duplication of statistics. For the same reason, in public security institutions, public security substations, police stations, police workstations, etc., are combined into one category. Stations and metro stations are used to represent the public transportation facilities of townships. Stations include bus and coach stations, and metro stations include light rail and subway stations. By distinguishing different places of the same type, the structural equation model can be best leveraged and the ecological fallacy can be avoided.

Limited by data availability, the population data for each township come from the Seventh National Census of China in 2020, the bulletin published closest to the study time.

Based on Baidu Encyclopedia, the administrative division area data of each township from January 16, 2022 is collected, and these data are verified using the area data of some townships officially released in the China County Statistical Yearbook (Township Volume) in 2018 and the area data estimated based on ArcGIS software, which found that the encyclopaedia data were accurate. Due to the limitations represented by the official data being incomplete and the software estimation data containing some errors, the densities of various facilities, premises, and populations are finally calculated based on Baidu Encyclopedia data.

In summary, all the data in the official administrative division of townships is collated for this paper. In the following, the hierarchical regression and structural equation models regarding townships are analysed.

### Regional characteristics

#### Thefts within the area

**Fig. 2:**
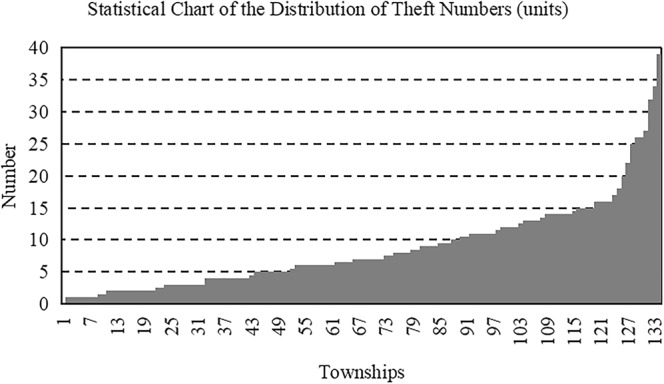
Statistical chart of the distribution of theft numbers. The number of thefts varies between townships.

A large number of thefts have occurred in the urban area of Beijing. In 134 townships, the occurrence of theft shows a state of cluster distribution, as shown in Fig. 2. Among them, the theft numbers in certain townships are significantly higher, which may be related to the unique environmental and social conditions in these regions. As a result, the aggregation of thefts in the research area is verified, providing a basis for accurately portraying the cold spots and hotspots of crime and the empirical analysis in the following paper.

**Fig. 3:**
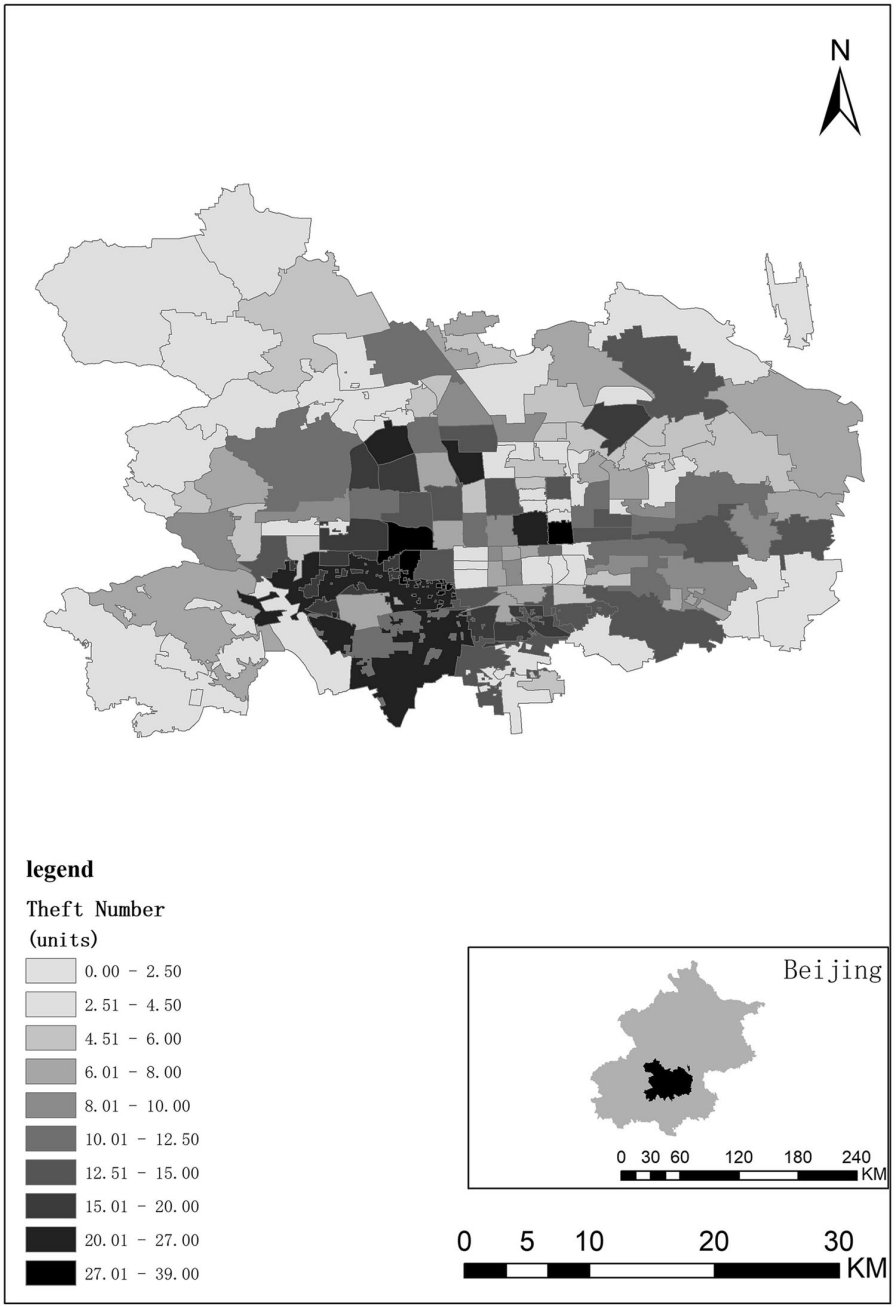
Theft hotspot map at the township level. Theft numbers in Beijing have hotspots in terms of space.

When thefts are spatially clustered and distributed, the incidence of theft forms both hotspots and cold spots. Based on ArcGIS software, this paper draws a map of theft hotspots at the township level and intuitively displays the differences in theft numbers in various administrative regions in Fig. 3. In the urban area of Beijing, the regions with the highest theft numbers are concentrated in the central area and southwest, and crime hotspots are there. At the same time, based on ArcGIS software, this paper calculates the spatial autocorrelation index of theft numbers in the urban area and obtains the following values: Moran *I* index = 0.059218, *Z*-score = 2.759186, and *P*-value = 0.005795. There is a significant spatial positive autocorrelation in the urban theft numbers, and the theft shows a significant clustered distribution.

#### Various study factors in the area

**Fig. 4:**
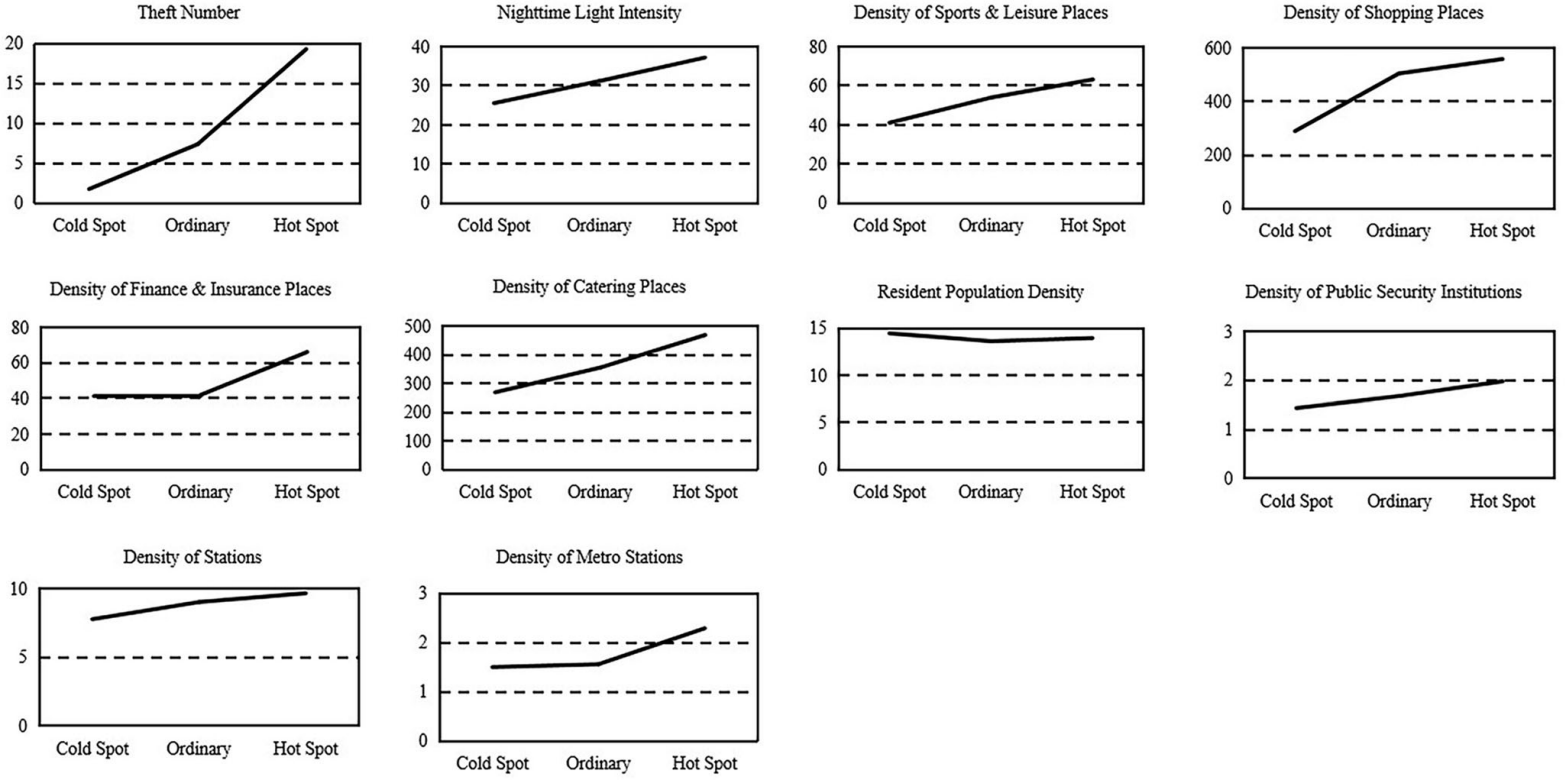
Means of theft number and various factors. Theft number and various factors have significant differences in hot, ordinary, and cold spots.

Among the 134 townships, this paper divides the 27 townships whose crime numbers are in the top 20% as hotspots and the 27 townships whose crime numbers are in the bottom 20% as cold spots and displays the means of the theft numbers and various factors in the hotspots, ordinary regions and cold spots in Table 2. For ease of comparison, the ten line charts drawn from various means are shown in Fig. 4.

**Table 2:** List of the means of theft number and various factors (*N* = 134).

Region type	TD	NI	SL	SP	FI	CA	PD	PS	BS	MS
Cold Spot	1.81	25.58	41.14	289.62	41.22	268.64	14.45	1.44	7.78	1.50
Ordinary	7.42	31.22	54.01	504.68	41.91	355.01	13.62	1.68	9.03	1.56
Hot Spot	19.33	37.20	63.21	558.65	66.14	469.03	13.95	1.98	9.67	2.29

*N* sample size, *TD* theft, *NI* nighttime light intensity, *SL* sports and leisure places, *SP* shopping places, *FI* finance and insurance places, *CA* catering places, *PD* resident population, *PS* public security institutions, *BS* station, *MS* metro station.

As seen in Fig. 4, the nighttime light intensity, the densities of various commercial premises, the density of public security institutions, and the densities of various public transport facilities are significantly higher in hotspots than in cold spots. In contrast, the density of the resident population is relatively lower in hotspots than in cold spots. This may be explained as follows:

(1) As stated by the RAT, various commercial premises and public transport facilities serve as key nodes in the routine activities of both city dwellers and potential offenders and thus lead to high theft rates (see sections ‘Commerce and thefts’ and ‘Other factors and thefts’ for details).

(2) The degree of commercial prosperity as represented by the nighttime light intensity is the social factor and neighbourhood characteristic that SDT most focuses on. At the same time, according to RCT theory, a higher degree of commercial prosperity can encourage potential thieves to be attracted to a township because they might believe that it offers more profit opportunities.

(3) According to the SDT and specific situations of Beijing, the higher that the resident population density is, the stronger the informal social control, thus providing more crime regulations. This explains the higher density of the resident population in theft cold spots, meaning that the relationship between thefts and the resident population may be negative.

(4) As described in section ‘Other factors and thefts’, traditional criminology proposes, from the perspective of “deterrence,” that the presence of police agencies can suppress crimes. In Chinese environmental criminology studies, scholars have observed that the mean of public security institutions’ densities in hotspots is much higher than in ordinary areas and cold spots and explained this phenomenon through the following: “enhancing policing strength is a natural response to high crime rates” and “the ability of police agencies to suppress crimes is relatively limited near crime hotspots” (Liu and Zhu, 2016; Liu et al., 2019; Shan, 2020). This means that the relationship between thefts and police agencies may be positive.

Statistical analysis models are then established to test these theoretical speculations.

## Results

### Research results based on the hierarchical regression model

**Fig. 5:**
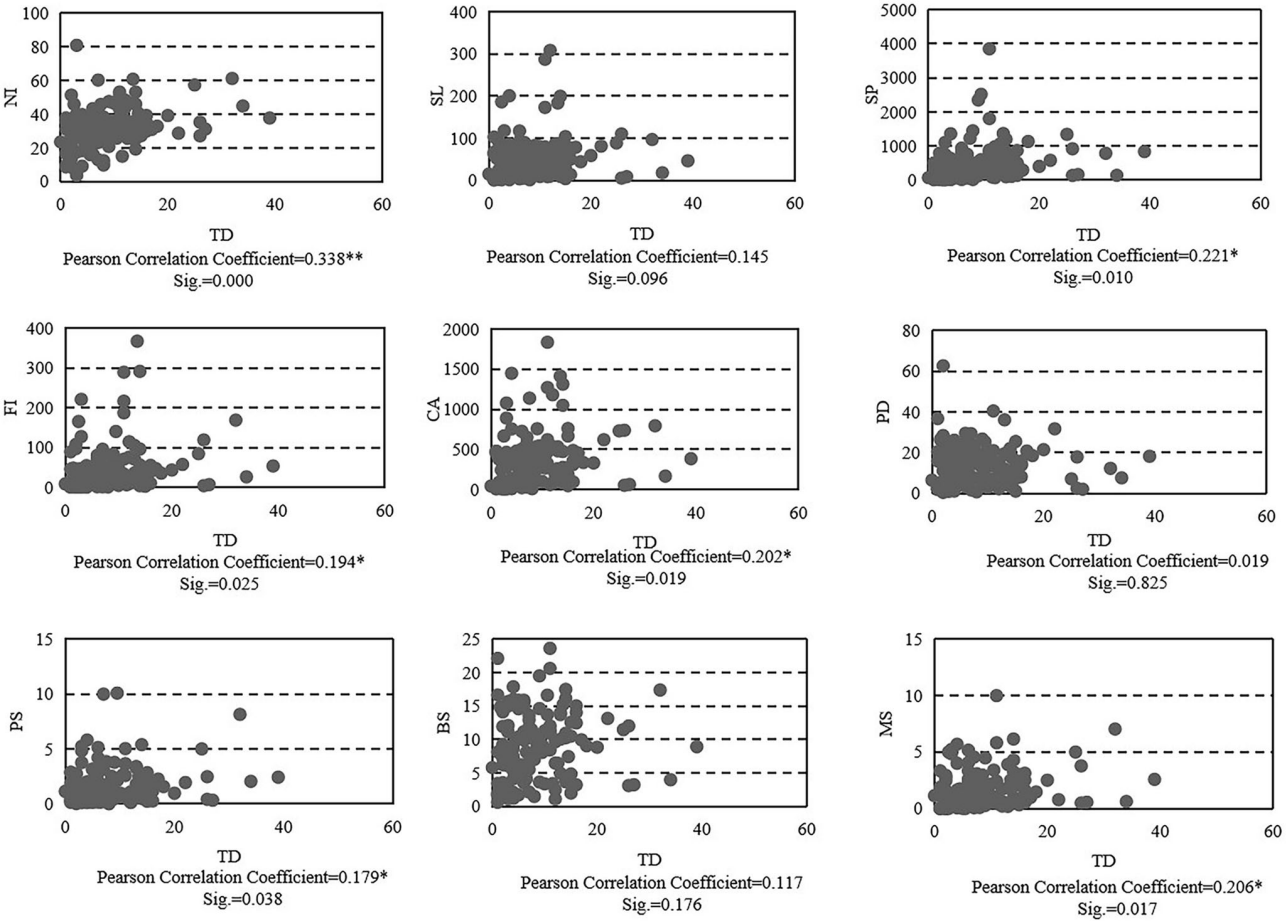
Correlation analysis results. TD theft, NI nighttime light intensity, SL sports and leisure places, SP shopping places, FI finance and insurance places, CA catering places, PD resident population, PS public security institutions, BS station, MS metro station, Sig. significance.

Based on SPSS software, every data point and TD were analysed using two-factor correlation analysis and bilateral significance tests. Figure 5 displays the Pearson correlation coefficient, significance, and bivariate scatter plots. Multiple commercial premises factors and the nighttime light factor are significantly related to thefts. In other words, these factors may improve the ability of regression models to explain theft. Therefore, it is necessary to establish a hierarchical regression model to place these two types of factors at different levels and observe their roles and effects in explaining thefts.

A hierarchical regression model based on multiple linear regression is designed as the independent variable with each data point and TD is defined as the dependent variable (Table 3). This paper introduces the relevant data of the resident population, public security institutions, public transportation facilities, and commercial premises as contained in Models 1–4, each in turn, and finally introduces the most critical observed factor, nighttime light intensity, and establishes the final Model 0. Accordingly, this paper observes five types of factors at different levels, highlighting their different effects on the dependent variable.

**Table 3:** Analysis results of the hierarchical regression model (*N* = 134).

Variable	Model 1		Model 2		Model 3		Model 4		Model 0	
	Coe.	Sig.	Coe.	Sig.	Coe.	Sig.	Coe.	Sig.	Coe.	Sig.
Intercept	8.501	0.000	8.033	0.000	7.810	0.000	7.966	0.000	3.718	0.038
PD	0.014	0.825	−0.055	0.423	−0.090	0.271	−0.103	0.222	−0.094	0.245
PS			0.837	0.027	0.444	0.316	0.217	0.647	−0.083	0.858
BS					0.011	0.953	−0.028	0.891	−0.122	0.536
MS					0.750	0.158	0.355	0.546	0.229	0.687
SL							−0.026	0.385	−0.032	0.269
SP							0.002	0.391	0.002	0.320
FI							0.007	0.663	0.000	0.998
CA							0.005	0.389	0.003	0.543
NI									0.216	0.001
*R*^2^	0.000		0.037		0.058		0.081		0.154	
*F*	0.049		2.512		1.975		1.379		2.514	
Sig.	0.825		0.085		0.102		0.212		0.011	
∆*R*^2^			0.037		0.021		0.023		0.073	

*N* sample size, *Coe*. coefficient, *Sig*. significance.

Note: (1) This paper aims to judge the interpretation ability of independent variables in each level on the dependent variable by the change of *R*^2^ based on the hierarchical regression model rather than assume the joint influence of all independent variables on the dependent variable, so the requirements for *R*^2^ are more relaxed.

(2) In the section on *Study variables and data sources*, this paper verifies that there are no significant unacceptable correlations between variables through collinearity diagnostics. It does not mean no relationships between these variables, as shown in the structural equation model. Still, it statistically guarantees the rationality of the hierarchical regression model.

In introducing variables at different levels, the relevant variables of public security institutions and public transportation facilities increased R^2^, improving the model’s explanatory ability. The introduction of the relevant variables regarding commercial premises also improves the explanatory ability of the model. That is, introducing commercial environmental factors enhances the model’s ability to explain the theft phenomenon. This demonstrates the effectiveness and applicability of using environmental criminology theories in explaining the impact of commerce on theft in Beijing. After introducing nighttime light intensity, the Δ*R*^2^ becomes the largest of the four values. That is, introducing a commercial social factor significantly enhances the model’s ability to explain the theft phenomenon. This means that theories such as SDT, which view commerce as a social factor, can provide practical assistance in explaining the theft phenomenon, so introducing this factor is extremely valuable. Therefore, the model results validate certain theoretical assumptions and support the construction of the structural equation model.

In addition, the significance of the individual variable shows that the relationship between NI and TD is significant, which also verifies the vital value of NI data in explaining thefts. It is worth mentioning that the relationships between other individual variables and TD are nonsignificant, so Model 0, which is a model based on multiple linear regressions, is difficult to apply to the comprehensive analysis of thefts in Beijing. Therefore, a structural equation model is later introduced to reflect Beijing’s theft problem more thoroughly.

### Research results based on the structural equation model

#### The fitting degree

Based on the conceptual analysis model constructed above, this paper uses Amos software and various data to fit and evaluate the model. The fitting indices of the structural equation model are displayed in Table 4. As seen from the table, each fitting index is within the acceptable range, and the fitting results of the model are good.

**Table 4:** Fitting indices of the model (*N* = 134).

	CMIN/DF	*P*	GFI	CFI	TLI
Default model	4.723	0.000	0.835	0.875	0.824

*N* sample size, *CMIN* the minimum value of the discrepancy, *DF* the degrees of freedom, *P* probability, *GFI* the Goodness of Fit Index, *CFI* the Comparative Fit Index, *TLI* the Tucker-Lewis Coefficient.

#### The measurement results of the model

**Fig. 6:**
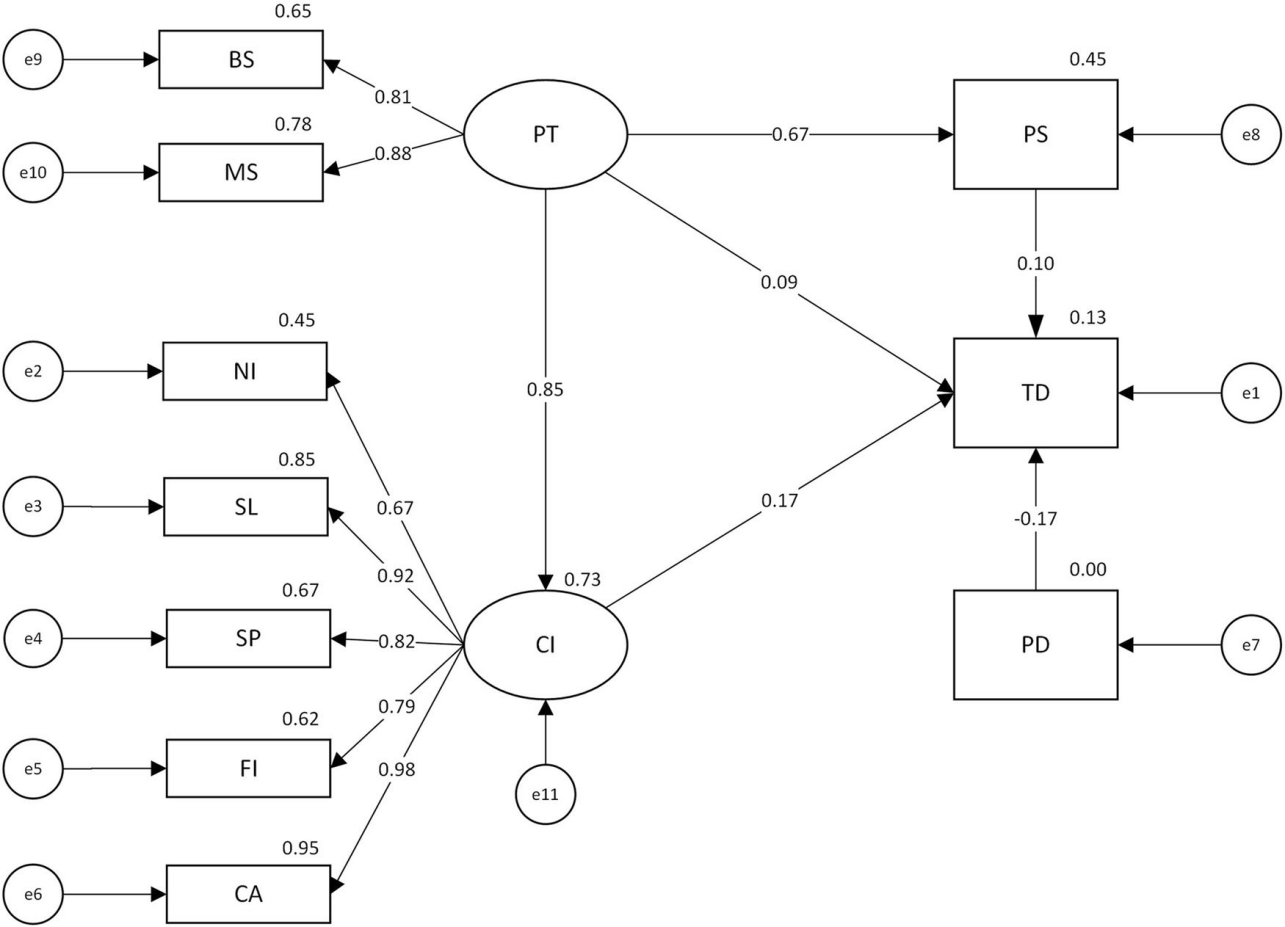
Measurement results of the model. BS station, MS metro station; PT public transport facilities, NI nighttime light intensity, SL sports and leisure places, SP shopping places, FI finance and insurance places, CA catering places, CI commerce, PS public security institutions,TD theft, PD resident population.

This paper shows the model’s measurement results and standardised path coefficients in Fig. 6.

Among them, the factor loads of the two observed variables for public transportation facilities are greater than or equal to 0.81, which indicates that they are strong indicators of public transportation facilities and that the factor selection in this paper is reasonable. The factor loads of all four commercial environmental variables are greater than or equal to 0.79, indicating that they are all strong commerce indicators. We can see that commercial environmental factors can effectively be used to characterise commerce. The commercial social variable of “NI” has a factor load of 0.67, which meets the statistical requirements and indicates that it is a helpful indicator for representing commerce. Combined with the hierarchical regression model results, it is argued in this paper that this commercial social factor can be used to effectively characterise commerce. The use of two commercial variables, then, can effectively describe the commerce phenomenon in Beijing.

#### Variables’ relationships

The path test results of the structural equation model are displayed in Table 5.

**Table 5:** Path test results (*N* = 134).

Path	Non-standardised coefficients	Standardised coefficients	S.E.	C.R.	*P*
PT → TD	0.161	0.092	0.419	0.384	0.701
PT → PS	0.293	0.673	0.036	8.103	***
PS → TD	0.387	0.096	0.476	0.814	0.416
PD → TD	−0.123	−0.167	0.059	−2.068	0.039
PT → CI	68.552	0.854	6.500	10.547	***
CI → TD	0.004	0.167	0.004	0.842	0.400

*N* sample size, *S.E*. standard error, *C.R*. the regression weight divided by the standard error, *P* the probability.

*PT* public transport facilities, *TD* theft, *PS* public security institutions, *PD* resident population, *CI* commerce.

***The regression weight for PT in the prediction of PS or CI is significantly different from zero at the 0.001 level (two-tailed).

As seen from the table:

(1) There is an nonsignificant positive relationship between PT and TD, a significant positive relationship between PT and PS, and an nonsignificant positive relationship between PS and TD. Public transportation facilities provide nonsignificant convenience for theft and thus attract more policing as a crowded hub. The positive relationship between policing and theft is consistent with previous findings from China (Liu and Zhu, 2016; Liu et al., 2019; Shan, 2020).

(2) There is an nonsignificant positive relationship between CI and TD and a significant positive relationship between PT and CI. Public transportation facilities drive commercial activities in the region, and commerce attracts thefts to a certain extent.

(3) There is a significant negative relationship between PD and TD at the 5% level. Beijing’s resident population effectively increases the level of informal social control to curb theft.

The above results (especially the positive and negative effects of influence) corroborate the conceptual analysis model and research hypotheses presented in section ‘Research hypotheses’. After verifying the correctness of the conceptual model, the impact of Beijing’s commerce on theft can be explored based on the relationship between the latent variable of CIs and the latent variable of TD as highlighted by the structural equation model. In addition, the following points need to be made: In Models 2–4, Model 0, and the structural equation model, PD and TD are negatively related, which indicates their relationship’s credibility. However, the relationship between PS and TD is significantly positive in Model 2, nonsignificantly positive in Models 3 and 4, and nonsignificantly negative in Model 0. Since the positive or negative nature of this relationship varies in relatively perfect models and is not significant, it is difficult to judge the positive or negative nature of this relationship based on the results of the hierarchical regression model. It is precisely because multiple univariates are not significant in the hierarchical regression model that a structural equation model is constructed, as it better aligns with actual situations and statistical requirements. Since the hierarchical regression model cannot be used to verify the relationship between PS and TD in the structural equation model, the relationship between the two cannot be judged based on the statistical results but rather relies on the existing results to verify that this positive relationship has a certain degree of credibility (Liu and Zhu, 2016; Liu et al., 2019; Shan, 2020).

### Discussion

First, commerce does not significantly facilitate theft in Beijing. After verifying the effectiveness of the commercial environmental and social variables in explaining theft numbers using the hierarchical regression model, a structural equation model is constructed that is more realistic, more statistically satisfactory and more comprehensively analyses commerce’s influence on theft with the help of the latent variable of commerce. The relationship between the two is nonsignificant, as seen from the statistical results regarding CI → TD. The higher factor loads of commercial environmental variables in the structural equation model and the nonsignificant relationships between them, as well as the dependent variable in Model 0, can arguably be used to verify this relationship. In other words, the commercial environmental variables can better reflect Beijing’s commerce than the commercial social variables, and their positive impact on theft is minimal, so the promotion effect of Beijing’s commerce on theft is not significant overall.

Second, two kinds of commercial variables and their corresponding theories, namely, environmental criminology and SDT, can be used to effectively explain commerce’s impact on theft in Beijing. In the hierarchical regression model based on multiple linear regression, the introduction of commercial environmental and social variables improves the explanatory ability of the model, which means that commercial environmental and social variables can both be used to explain theft numbers and identify theft hotspots. In the structural equation model, the factor loads of the five observed variables of the commercial environmental and social categories are greater than or equal to 0.67, which shows that they are ideal indicators for representing commerce. Therefore, this paper argues that these two types of variables and their corresponding theories can be used to effectively explain the relationship between commerce→theft in Beijing.

## Conclusions

Commerce is the primary and essential dynamic in studying theft causes and has a complex relationship with theft. Hence, commerce’s impact on theft yet needs to be further verified, especially in non-Western contexts. Given that classical theories such as environmental criminology and SDT mostly suggest that commerce can facilitate theft, this paper uses hierarchical regression and structural equation models to analyse the relationship between commerce and theft in Beijing and verifies the applicability and validity of the two types of commercial variables and classical Western-origin theories in explaining Beijing’s phenomenon. This paper reaches the following conclusions:

First, commerce does not significantly promote theft in Beijing. Given the limited effectiveness of Model 0 in the hierarchical regression model in explaining the combined impact of commerce on thefts, this paper constructs a structural equation model that is more in line with actual situations and statistical requirements. In the latter, a nonsignificant positive impact of commerce on theft can be detected, which can be verified to some extent by the former. Second, both types of commercial variables and classical theories, such as environmental criminology and SDT, are valid and applicable in interpreting the relationship between commerce and theft in Beijing. According to the hierarchical regression model, introducing commercial environmental and social variables improves the explanatory ability of the model. According to the structural equation model, both kinds of observed variables have high factor loads, i.e., factor loads of greater than or equal to 0.67, effectively representing commerce. Therefore, these two types of variables and the corresponding classical theories are essential in explaining commerce’s impact on theft in Beijing.

This paper has three main limitations. First, in terms of data selection, due to the limitations of data availability and the tendency to conduct research based on whole years, this paper uses only one year of theft data, so it is difficult to determine whether fluctuations occur between years. Due to the time lag involved in the discovery of crimes and the lack of information in the judgement documents, this paper fails to extract accurate and sufficient data on the time of thefts, so the time factor cannot be sufficiently accounted for. Second, in terms of method selection, although the main structural equation model in this paper solves some problems with traditional research methods, it also has some limitations, such as its assumption that all relationships are linear (Najaf et al., 2018). Third, this paper examines ordinary theft in Beijing and does not verify the model’s applicability in two specific areas of theft, namely, burglary and motor vehicle theft.

Based on the above limitations, this paper proposes some potential directions for improvement for future research. First, the study period can be extended and the time factor considered. Second, the fixed relationship assumption problem of the structural equation model can be solved and the nonlinear function form can be introduced into the model design, further developing the model built in this paper. Third, the model can be verified or modified by ascertaining the applicability of the model constructed in this paper to the study of other types of theft.

## Data Availability

The data supporting this study’s findings are available on request from the corresponding author.

## Footnotes

1 According to the Law Yearbook of China, property fraud increased from 29.49% in 2019 to 40.07% in 2020, an overall proportion statistic that does not distinguish between categories of property fraud. According to the analysis of crime trends in the “Blue Book of Chinese Crime Governance (2020),” this phenomenon is related to the grim situation of cybercrimes. In 2020, the theft of personal information was frequent, promoting targeted telecom fraud in emails, text messages, etc.

2 Commerce is an organised activity that provides goods and services required by consumers and is an important part of urban functions. Commerce can be seen both as an activity that enables the circulation of goods and services in specific places and as an urban cultural phenomenon in social development. Therefore, this paper uses two types of factors to comprehensively characterise commerce in Beijing, namely, social and commercial environmental factors.

3 China adopts a dualistic approach to addressing theft, dividing related cases into criminal and public security cases. Criminal theft cases are comprehensively defined in a quantitative and qualitative manner based on the involved amount and the case nature, account for 43.90% of all theft cases accepted by public security organs nationwide in 2020, seriously impact society and are of great research value.
